# Supplementary material for: Current smoking alters phospholipid- and surfactant protein A levels in small airway lining fluid: An explorative study on exhaled breath
Source: PLoS One. 2021 Jun 25;16(6):e0253825. doi: 10.1371/journal.pone.0253825 (PMC8232447; doi:10.1371/journal.pone.0253825)
Supplement: S1 File — (PDF) [file pone.0253825.s001.pdf]

ID	Sex	Age	ht	wt	bmi	packyears	smoking	NO50_ppb	crp	pex_ng_breath	pex_ng_L	knL	knbreath	Nr_breath	Corr_vol	DPPC_wt_PEX	POPC_wt_PEX	SPA_wtPEX	Alb_wtPEX	meanSIII_pN2_L	fvc_L	fev1_L	fev1_fvcperc	fvc_L_post	fev1_L_post	fev1_fvcperc_post
1	0	69,0	176	96	31,0	13,0	1	11,0	0,51							9,2	2,4	2,5	8,0	1,4	3,7	2,9	78,3	3,8	3,0	79,0
2	1	67,0	159	63	24,9	50,0	2	7,0	3,1	48,0	21,3	77,9	175,4	6	13,5	11,8	3,7	7,0	15,3	4,2	2,8	2,0	71,6	3,0	2,1	72,2
3	1	52,0	165	75	27,5	30,0	2	17,0	3,9	7,8	2,7	10,6	30,6	16	46,1			1,6	4,0	1,4	3,1	2,4	78,0	3,1	2,5	80,3
4	0	52,0	181	87	26,6	0,0	0	17,0	9,7	7,6	2,8	7,9	21,4	16	43,3	6,1	1,6	1,7	8,6	1,3	5,2	3,9	75,3	5,2	4,1	78,1
5	1	47,0	168	84	29,8	0,0	0	22,0	0,4	22,7	9,4	36,7	88,9	6	14,5	10,4	3,6	3,8	5,0	1,3	3,4	2,7	79,7	3,3	2,7	82,2
6	0	76,0	173	74	24,7	29,0	2	20,0	1,3	3,9	1,7	6,0	14,3	14	33,2			2,0	6,8	2,8	3,5	2,0	58,1	3,8	2,8	73,0
7	0	73,0	175	72	23,5	25,0	1	40,0	3,7	19,4	5,9	17,1	56,6	7	23,2	8,9	2,4	3,2	10,1	1,0	4,6	2,9	62,4	4,8	3,1	65,5
8	0	72,0	171	73	25,0	3,0	1	25,0	1,1	2,8	1,3	4,5	9,8	35	75,9	12,8	3,9	3,2	8,9	5,3	3,0	1,9	64,5	3,2	2,1	65,1
9	1	70,0	153	61		34,0	1	80,0	1,6	10,3	2,0	5,5	28,8	12	63,1	11,6	2,9	2,1	5,6		2,1	1,6	74,5	2,2	1,6	70,7
10	0	72,0	182	99	29,9	25,0	1	22,0	1,4	13,5	7,2	34,6	64,9	9	16,9	10,8	3,5	2,4	1,9	2,5	4,2	2,8	65,2	4,5	3,0	68,0
11	0	72,0	167	68	24,4	46,0	1	19,0	1,6	21,2	8,7			6	14,6	9,3	2,5	2,5	9,7	5,3	2,8	1,9	69,5	3,0	2,2	73,6
12	0	71,0	171	74	25,3	21,0	1	21,0	1,7	12,8	2,5	7,3	36,6	10	50,3	10,7	2,4	2,4	9,4	4,4	3,6	2,5	67,3	3,7	2,6	70,8
13	0	71,0	171	57		16,0	1	16,0	0,3	27,8	10,2	43,0	116,8	5	13,6	11,0	3,2	3,4	6,5		3,5	2,8	79,7	3,4	2,8	81,8
14	0	71,0	170	76	26,3	38,0	1	17,0	1,4	12,0	5,9	23,2	46,9	11	22,2	12,0	3,5	4,5	7,2	1,9	3,1	2,4	77,4	2,9	2,3	77,1
15	1	70,0	178	89	28,1	10,0	1	13,0	0,8	8,7	3,0	11,1	32,5	15	43,9	11,8	3,0	4,2	11,1	3,2	3,7	2,7	71,3	3,7	2,8	76,2
16	1	69,0	160	74	28,9	0,0	0	21,0	0,8	14,4	6,2	20,5	47,7	9	21,0	7,2	2,3	2,8	3,7	4,7	2,6	1,6	59,5	2,7	1,8	66,9
17	1	69,0	166	46	16,7	44,0	2	12,0	0,5	12,1	4,4	16,7	45,4	10	27,3	12,0	5,0	4,2	6,4	3,4	3,2	2,5	77,1	3,2	2,6	79,6
18	0	68,0	176	93	30,0	48,0	1	89,0	0,6							10,5	2,8	3,5	10,4	4,2	3,4	2,6	75,7	3,9	2,9	75,1
19	1	66,0	158	57	22,8	50,0	2	22,0	1,2	8,9	5,6	19,1	30,5	14	22,4	10,2	2,7	2,8	2,8	12,0	2,3	1,5	64,4	2,4	1,5	64,8
20	1	66,0	165	77	28,3	4,0	1	24,0	5,2	6,2	2,4	9,1	23,3	16	41,1	6,7	1,8	2,7	5,9	5,5	2,6	1,4	52,7	2,8	1,6	57,0
21	0	66,0	183	124	37,0	36,0	1	10,0	2,8	9,1	2,8	10,4	33,4	14	45,2	11,3	3,6	3,1	8,5	2,4	4,0	2,9	71,8	4,0	3,1	79,0
22	0	66,0	187	77	22,0	15,0	2	20,0	3,0	16,5	3,2	9,4	48,6	8	41,2	9,5	2,9	4,2	9,8	0,4	5,9	4,0	68,4	5,8	4,0	68,8
23	1	66,0	164	61	22,7	23,0	2	11,0	0,9	27,7	13,1			6	12,6	13,3	3,7	4,3	10,7	4,0	2,5	1,6	63,2	2,7	1,5	57,2
24	1	65,0	167	66	23,7	23,0	2	999,0	0,7	10,1	4,5	17,9	40,4	13	29,3	13,3	3,8	3,5	5,2	4,9	2,7	2,0	73,6	2,9	2,1	72,7
25	1	64,0	165	60	22,0	40,0	2	9,0	1,3	20,3	8,3	26,7	65,2	6	14,7	12,0	3,6	4,1	6,0	4,7	3,4	2,2	66,6	3,4	2,3	67,6
26	0	64,0	182	62	18,7	50,0	2	16,0	0,5	14,0	4,4	13,4	42,6	9	28,5	17,9	5,1	5,0	13,6	1,3	4,2	3,2	76,2	4,1	3,1	75,8
27	0	64,0	172	105	35,5	32,0	1	25,0	5,4	14,0	6,0			9	21,1	10,6	3,5	3,4	11,7	1,5	3,5	2,9	83,6	3,6	2,9	81,0
28	1	64,0	145	66	31,4	1,5	1	19,0	3,1	6,7	5,9	22,5	25,6	18	20,5	5,3	1,8	2,2	6,0	4,0	1,6	1,0	61,5	1,6	0,9	60,3
29	0	64,0	182	89	26,9	33,0	1	12,0	2,0	13,9	5,7	17,0	41,7	9	22,0	10,0	3,3	3,9	8,7	1,2	3,7	3,0	80,4	3,7	3,1	83,5
30	0	62,0	191	76	20,8	30,0	1	11,0	0,7	16,1	4,6	14,4	50,2	8	27,9	14,0	3,9	4,8	8,8	0,7	4,9	3,6	73,0	4,9	3,6	73,2
31	0	61,0	184	92	27,2	18,0	2	23,0	2,8	8,9	4,1	13,0	28,4	14	30,6	10,7	3,1	3,6	6,0	1,1	5,6	3,9	69,1	5,6	4,1	73,2
32	1	60,0	161	65	24,9	30,0	1	13,0	0,5	7,8	2,9	10,8	29,5	16	43,8	12,1	4,2	3,2	8,4	1,3	3,8	3,4	89,4	3,4	2,7	81,5
33	0	59,0	170	81	28,0	42,0	2	28,0	6,2	10,0	3,3	9,7	29,3	12	36,2	15,1	3,8	1,4	5,6	4,0	3,7	2,4	63,9	3,9	2,5	63,9

34	1	59,0	158	96	38,5	52,0	1	19,0	6,2	5,8	3,1	13,7	25,6	22	41,2	10,0	2,9	4,1	5,3	15,1	2,0	1,7	84,0	1,9	1,6	83,4
35	0	58,0	190	80	22,2	0,0	0	19,0	3,0	7,3	2,0	6,6	24,5	17	63,4	9,7	2,8	3,1	4,0	0,4	5,9	3,7	62,7	5,8	3,8	65,8
36	1	58,0	170	60	20,8	28,0	2	7,0	0,2	5,8	1,7	5,5	19,0	21	73,3	12,4	4,2	4,3	11,0	1,4	4,2	3,2	76,7	4,2	3,3	77,3
37	1	57,0	162	78	29,7	45,0	2	10,0	1,7	51,0	25,9	89,9	177,2	6	11,8	10,8	3,6	1,4	4,6	2,0	3,2	2,5	77,8	3,1	2,5	79,9
38	0	56,0	184	76	22,4	20,0	2	21,0	1,8	19,0	8,0	23,0	54,8	8	19,1	9,3	2,7	5,1	8,3	1,5	5,6	3,9	68,8	6,0	4,2	68,8
39	1	55,0	169	70	24,5	4,0	2	14,0	0,9	18,9	8,0	29,1	68,4	7	16,5	12,6	4,5	2,2	6,9	1,9	3,8	2,8	74,7	3,8	2,9	76,0
40	0	54,0	186	84	24,3	40,0	2	19,0	6,9	44,7	16,0	56,4	157,4	6	16,8	10,3	3,7	5,1	5,9	1,8	5,3	2,9	55,0	5,3	3,1	58,1
41	0	52,0	168	77	27,3	22,0	1	23,0	0,9	8,8	3,2	13,5	36,8	14	38,3	11,6	3,7	3,8	8,8	1,7	4,1	2,8	69,5	4,2	3,1	73,2
42	0	51,0	177	105	33,5	14,0	2	24,0	4,5	9,0	2,4	10,0	36,9	14	51,9	11,3	4,0	4,9	12,4	0,9	5,2	3,6	69,7	5,2	3,6	69,6
43	0	50,0	180	91	28,1	36,5	1	10,0	1,0	16,6	5,6	14,5	43,2	8	23,8	7,7	2,2	2,0	12,2	1,4	5,1	3,2	63,8	5,1	3,2	63,8
44	1	49,0	159	60	23,7	22,0	1	12,0	0,4	7,6	2,9	9,3	24,6	16	42,3	10,4	3,2	2,9	1,9	2,1	3,6	2,5	68,9	3,6	2,6	72,0
45	1	48,0	170	81	28,0	12,0	2	17,0		25,0	13,5	49,7	91,9	6	11,1			1,8	5,4	2,5	3,1	2,3	73,5	3,1	2,4	76,8
46	1	47,0	167	76	27,3	14,0	2	9,0	0,9	10,0	4,9	19,4	39,8	12	24,7	11,0	4,0	3,8	7,6	1,1	3,0	2,3	75,8	3,2	2,5	77,2
47	1	75,0	164	80	29,7	35,0	1	6,0	1,0	15,3	3,7	11,3	47,0	8	33,4	11,6	2,8	5,0	9,9	3,7	3,3	2,4	71,7	3,3	2,4	71,1
48	0	72,0	185	82	24,0	10,0	1	16,0	0,3	9,6	3,6	12,7	33,8	13	34,5	10,9	3,4	4,0	11,2	1,0	4,5	3,3	72,7	4,4	3,4	77,9
49	1	70,0	166	73	26,5	20,0	1	33,0	1,2	8,6	4,0	18,9	40,7	14	30,2	11,4	3,2	3,7	9,9	6,2	2,5	1,9	75,1	2,6	2,0	78,2
50	1	83,0	156	51		999,0	1	21,0	0,6	15,0	9,1	36,3	60,1	9	14,9	10,5	3,7	2,3	8,9		2,1	1,6	78,6	2,1	1,7	80,6
51	1	81,0	163	64	24,1	46,0	1	69,0	1,1	13,0	5,6	20,1	46,9	10	23,3	5,5	1,5	2,7	6,8	3,5	2,7	1,8	65,7	2,9	1,8	62,2
52	0	83,0	161	56	21,6	0,0	0	11,0	1,3	12,3	4,2	11,3	33,0	10	29,3	11,4	4,3	3,6	22,0	3,4	3,5	2,1	60,5	3,6	2,2	61,4
53	0	81,0	177	84		999,0	1	30,0	2,9	14,5	5,8	22,1	55,0	9	22,4	14,5	4,3	4,2	30,3		3,2	2,0	63,4	3,2	2,0	63,0
54	0	79,0	190	92	25,5	71,0	1	26,0	0,7	3,4			6,9	37		6,9	1,7	1,7	7,7	5,3	3,3	1,6	48,6	3,9	2,0	49,7
55	1	75,0	170	73	25,3	0,0	0	14,0	3,2	6,7	3,6	13,8	25,4	18	33,1	13,1	5,0	3,8	7,5	4,0	2,4	1,6	66,4	2,4	1,7	69,3
56	0	74,0	177	68	21,7	49,0	2	26,0	3,2	10,3	4,7	9,6	21,0	12	26,2	9,0	2,1	4,3	5,4	4,8	3,2	1,6	52,1	3,8	1,8	47,5
57	0	73,0	176	69	22,3	55,0	2	19,0	6,1	19,4	10,3	22,3	42,0	7	13,2	9,9	2,4	4,1	7,8	4,6	3,7	2,6	71,7	3,8	2,7	71,4
58	0	67,0	178	82	25,9	0,0	0	11,0	0,8	4,3	1,2	4,8	17,7	28	102,5	10,8	2,8	3,2	10,5	1,6	3,5	2,3	64,9	3,6	2,5	69,1
59	0	71,0	169	71	24,9	28,0	2	16,0	1,7	35,5	17,9			4	7,9	10,6	4,3	2,9	10,1	2,3	3,1	2,2	70,7	3,1	2,2	71,1
60	1	70,0	174	121	40,0	20,0	1	17,0	5,2	14,7	6,3			9	20,8	8,6	2,0	2,3	8,3	4,7	2,5	1,6	65,3	2,6	1,7	66,4
61	0	70,0	184	93	27,5	22,0	1	16,0	0,9	10,0	4,4	15,9	35,8	13	29,4	13,5	3,6	2,2	4,2	0,5	4,7	3,5	74,7	4,7	3,7	77,5
62	0	69,0	175	96	31,3	20,0	2	12,0	1,6	34,6	11,9	31,5	91,2	5	14,5	10,9	3,6	3,9	9,2	2,0	3,7	2,4	64,7	3,9	2,5	65,7
63	0	68,0	188	110	31,1	38,0	2	14,0	2,7	58,0	13,2			3	13,2	15,7	6,2	7,8	17,7	2,4	4,0	2,7	67,2	4,0	2,8	71,4
64	1	68,0	158	68		0,0	0	18,0	2,3							7,6	2,1	1,9	5,0	1,4	2,7	2,2	79,0	2,5	2,1	83,6
65	0	67,0	182	73	22,0	999,0	2	24,0	1,0	31,5	8,9	34,1	121,4	4	14,2	14,8	4,5	6,5	12,9	3,0	4,2	2,8	65,4	4,3	2,9	67,7
66	0	67,0	181	81	24,7	0,0	0	38,0	1,5	5,2	1,7	5,4	16,8	23	72,0	5,9	2,0	2,5	8,2	0,9	4,5	3,3	73,3	4,7	3,5	75,3
67	1	67,0	166	81	29,4	0,0	0	17,0	2,5	12,4	5,4	18,5	42,2	10	22,9	9,2	2,2	1,8	7,7	0,6	3,3	2,6	78,4	3,2	2,5	79,0
68	0	66,0	168	75		0,0	0	20,0	0,7	21,4	5,8	23,8	87,7	6	22,2			3,3	13,2		4,2	3,5	83,5	4,3	3,7	84,5
69	1	64,0	168	93	33,0	31,0	1	16,0	8,1	3,1	1,3	4,8	11,4	38	89,9	7,4	2,5	1,8	6,4	4,0	2,4	1,8	76,2	2,5	1,8	73,1
70	0	63,0	177	76	24,3	0,0	0	23,0	0,8	15,3	4,8	14,4	45,9	8	25,5	8,1	2,5	2,7	8,6	1,9	4,6	2,9	64,5	4,4	3,2	73,2

71	0	61,0	167	68	24,4	40,0	2	13,0	1,2	14,8	4,6	15,4	49,8	9	29,1	10,2	3,3	4,5	15,5	1,4	4,1	3,0	74,1	3,9	3,1	78,2
72	0	62,0	182	91	27,5	36,0	2	18,0	1,3	30,6	8,9	33,9	116,6	5	17,2	11,7	3,9	1,8	6,9	0,9	4,7	3,7	79,0	4,5	3,7	80,8
73	0	61,0	185	84	24,5	9,0	1	19,0	1,6	28,8	6,2	20,4	94,5	5	23,2	12,0	3,9	5,1	12,4	0,8	5,2	3,4	66,5	5,1	3,5	69,9
74	1	58,0	169	80		999,0	2	23,0	2,1	23,6	8,8	52,7	141,5	5	13,4	16,9	4,2	5,9	8,6		2,8	1,9	67,6	2,8	2,0	73,3
75	1	58,0	162	66	25,1	5,0	1	12,0	1,5	21,7	9,5	24,1	55,3	7	16,1	8,9	2,7	3,4	11,0	3,0	3,3	2,1	64,0	3,3	2,2	66,2
76	1	56,0	176	78	25,2	20,0	2	30,0	1,4	9,5	3,5			13	34,9	16,7	5,2	4,1	8,9	1,8	4,2	3,2	75,8	4,2	3,2	76,7
77	0	55,0	189	97	27,2	0,0	0	17,0	1,4	10,0	2,1	8,6	41,0	12	57,0	17,2	5,6	3,7	10,3	0,7	4,8	3,3	68,2	5,0	3,5	70,3
78	1	55,0	170	73	25,3	31,0	2	11,0	2,6	6,7	3,0	11,8	26,2	18	39,8	12,8	4,3	2,5	4,2	3,5	2,9	2,0	70,6	2,6	2,1	79,2
79	1	54,0	161	73	28,2	33,0	2	5,0	14,0	41,5	18,1	78,0	178,9	6	13,8	9,8	3,3	3,3	6,3	1,3	2,5	2,1	84,0	2,4	2,1	85,8
80	1	53,0	174	71	23,5	20,0	1	14,0	0,6	11,2	4,2	13,1	34,8	11	29,1	7,0	1,5	3,8	10,7	2,6	4,1	2,4	59,5	4,1	2,7	64,5
81	1	52,0	167	85	30,5	0,0	0	21,0	1,9	13,4	4,4	16,6	51,0	9	27,7	11,7	3,2	4,3	11,1	1,4	3,5	2,4	67,1	3,7	2,6	69,6
82	0	52,0	183	76	22,7	0,0	0	32,0	0,5	4,9	2,8	6,1	10,9	27	47,9	7,1	1,8	1,3	5,6	0,8	5,3	3,9	73,8	5,2	4,0	77,5
83	0	51,0	168	74	26,2	0,0	0	28,0	0,4	19,3	4,8	20,2	82,0	7	28,4	12,9	4,4	2,9	6,5	0,8	4,8	4,0	82,7	4,6	3,9	83,6
84	1	50,0	160	59	23,0	14,0	2	11,0	1,4	20,7	11,1	37,3	69,5	6	11,2	11,5	3,4	3,0	5,4	0,7	3,8	3,0	77,5	3,8	3,0	78,3
85	1	50,0	166	69	25,0	0,0	0	16,0	1,0	21,0	6,6	22,9	72,5	7	22,2	12,0	3,0	3,1	7,2	1,2	3,7	2,8	76,3	3,6	2,9	80,3
86	1	50,0	166	54	19,6	0,0	0	19,0	0,2	8,3	2,7	10,5	32,0	15	45,5	8,7	2,6	2,5	6,7	1,0	3,7	3,2	85,4	3,8	3,2	84,6
87	1	50,0	164	69	25,7	0,0	0	23,0	0,5	31,3	9,1	36,5	125,6	6	20,7	10,3	2,9	3,8	8,7	0,7	3,8	2,9	76,3	3,7	3,0	80,2
88	1	49,0	164	67	24,9	0,0	0	34,0	0,9	20,7	8,0	26,9	69,3	6	15,4	11,7	5,0	3,1	6,1	0,6	3,1	2,6	84,5	2,9	2,5	86,1
89	1	47,0	175	60	19,6	0,0	0	28,0		8,1	2,8	9,2	27,2	15	44,1			2,5	5,2	1,7	3,7	2,3	61,0	4,0	2,6	66,2
90	1	48,0	169	68	23,8	0,0	0	16,0	2,0	16,1	6,0	18,3	49,1	8	21,5	8,5	2,5	2,0	4,3	2,5	3,7	3,0	80,2	4,0	3,2	80,4
91	0	46,0	180	84	25,9	25,0	2	13,0	0,6	18,6	8,0	19,8	46,0	7	16,3	8,3	2,0	1,8	6,0	1,4	5,7	4,4	77,1	5,4	4,3	78,6
92	0	46,0	191	93	25,5	0,0	0	18,0	1,5	7,2	2,0	7,5	26,6	17	60,0	9,0	2,7	2,3	7,5	1,5	5,2	3,4	66,3	5,2	3,6	69,4
93	1	44,0	170	70	24,2	18,0	2	13,0	4,7	53,3	18,6	71,1	204,0	3	8,6	4,5	1,6	4,4	7,3	1,1	3,5	2,7	76,3	3,6	2,8	77,7
94	0	44,0	179	87	27,2	18,0	1	27,0	1,3	10,6	3,4	12,7	39,7	12	37,7	8,2	4,0	3,3	6,3	1,4	4,3	2,9	65,9	4,3	3,0	70,3
95	0	45,0	173	75	25,1	0,0	0	18,0	0,4	4,6	1,0	4,5	20,9	27	124,7	10,7	4,0	3,1	7,5	1,1	5,2	3,8	72,3	5,3	4,0	76,6
96	1	44,0	160	66	25,8	1,0	2	21,0	2,5	17,3	5,9	20,8	60,9	8	23,4	11,3	3,9	3,0	3,9	0,8	3,7	2,9	78,4	3,8	3,2	83,3
97	1	44,0	179	70	21,8	0,0	0	15,0	0,3	44,7	15,1	50,9	150,8	6	17,8	10,4	3,5	3,3	6,1	1,0	4,5	3,3	74,0	4,4	3,4	78,0
98	1	43,0	170	67	23,2	8,0	2	7,0	1,5	14,2	7,4	28,5	54,8	9	17,3	10,8	3,5	2,7	5,3	0,7	4,0	3,4	86,1	4,0	3,5	88,0
99	1	43,0	172	61	20,6	0,0	0	23,0	1,0	8,8	2,5	13,2	45,4	14	48,3	9,3	3,0	3,0	4,6	1,4	4,2	3,2	75,1	4,0	3,2	79,8
100	0	42,0	184	80	23,6	0,0	0	12,0	1,1	7,1	1,5	7,8	36,3	17	79,3	11,9	10,3	2,3	6,0	0,6	5,3	4,0	74,6	5,2	4,2	80,0
101	0	39,0	185	95	27,8	0,0	0	30,0	0,7	9,6	2,2	8,2	36,5	13	57,7	9,1	2,7	3,2	9,1	0,9	5,1	3,7	72,2	5,3	4,0	75,5
102	0	66,0	185	89	26,0	0,0	0	46,0	1,1							15,4	3,9	3,9	8,9	1,3	5,2	3,1	59,6	5,1	3,2	63,4
